# Antibodies toward Na^+^,HCO_3_^–^-cotransporter NBCn1/SLC4A7 block net acid extrusion and cause pH-dependent growth inhibition and apoptosis in breast cancer

**DOI:** 10.1038/s41416-024-02591-0

**Published:** 2024-02-03

**Authors:** Trine V. Axelsen, Claus Olesen, Danish Khan, Ali Mohammadi, Elena V. Bouzinova, Christine J. F. Nielsen, Marco Mele, Katrine R. Hauerslev, Helene L. Pedersen, Eva Balling, Pernille Vahl, Trine Tramm, Peer M. Christiansen, Ebbe Boedtkjer

**Affiliations:** 1 Department of Biomedicine, Aarhus University, Aarhus, Denmark; 2 Department of Surgery, Randers Regional Hospital, Randers, Denmark; 3 Department of Plastic and Breast Surgery, Aarhus University Hospital, Aarhus, Denmark; 4 Department of Pathology, Randers Regional Hospital, Randers, Denmark; 5 Department of Pathology, Aarhus University Hospital, Aarhus, Denmark; 6 Department of Clinical Medicine, Aarhus University, Aarhus, Denmark

**Keywords:** Breast cancer, Metabolism, Target validation, Antibody therapy

## Abstract

**Background:**

Na^+^,HCO_3_^–^-cotransporter NBCn1/Slc4a7 accelerates murine breast carcinogenesis. Lack of specific pharmacological tools previously restricted therapeutic targeting of NBCn1 and identification of NBCn1-dependent functions in human breast cancer.

**Methods:**

We develop extracellularly-targeted anti-NBCn1 antibodies, screen for functional activity on cells, and evaluate (a) mechanisms of intracellular pH regulation in human primary breast carcinomas, (b) proliferation, cell death, and tumor growth consequences of NBCn1 in triple-negative breast cancer, and (c) association of NBCn1-mediated Na^+^,HCO_3_^–^-cotransport with human breast cancer metastasis.

**Results:**

We identify high-affinity (*K*_D_ ≈ 0.14 nM) anti-NBCn1 antibodies that block human NBCn1-mediated Na^+^,HCO_3_^–^-cotransport in cells, without cross-reactivity towards human NBCe1 or murine NBCn1. These anti-NBCn1 antibodies abolish Na^+^,HCO_3_^–^-cotransport activity in freshly isolated primary organoids from human breast carcinomas and lower net acid extrusion effectively in primary breast cancer tissue from patients with macrometastases in axillary lymph nodes. Inhibitory anti-NBCn1 antibodies decelerate tumor growth in vivo by ~50% in a patient-derived xenograft model of triple-negative breast cancer and pH-dependently reduce colony formation, cause G2/M-phase cell cycle accumulation, and increase apoptosis of metastatic triple-negative breast cancer cells in vitro.

**Conclusions:**

Inhibitory anti-NBCn1 antibodies block net acid extrusion in human breast cancer tissue, particularly from patients with disseminated disease, and pH-dependently limit triple-negative breast cancer growth.

## Introduction

The microenvironment of solid tumors is exceptionally acidic and hypoxic compared to most non-cancer tissue. Extracellular pH (pH_o_) can reach down to 6.5 in poorly perfused tumor regions [1, 2] with detrimental consequences for tissue structure [3] and the function of normal cells, including immune cells [4, 5]. Functional specialization allows cancer cells to survive harsh tumor conditions; [3, 6] and aspects of the microenvironment—such as elevated lactate concentrations—even appear to promote pathologic hyperplasia and treatment resistant cancer cell phenotypes [7, 8].

Upregulated net acid extrusion protects cancer cells against intracellular acidification, thereby maintaining conditions permissive for cell proliferation and metabolism [8]. Cellular net acid extrusion in human and murine breast cancer tissue occurs through a combination of Na^+^,HCO_3_^–^-cotransport and Na^+^/H^+^-exchange [3, 9–11]. Based on functional genomics approaches in mice, we previously showed that the electroneutral NBCn1, encoded by the *Slc4a7* gene, is predominantly responsible for the Na^+^,HCO_3_^–^-cotransport in breast carcinomas [9, 10]. Genome-wide association studies furthermore link variation in *SLC4A7* to breast cancer susceptibility in women of diverse ethnicities; [12–16] and we recently reported that the level of *SLC4A7* mRNA in human primary breast cancer biopsies predicts survival (hazard ratio 2.14–2.18) of women with luminal A or basal-like/triple-negative breast cancer [17].

Current pharmacological inhibitors of Na^+^,HCO_3_^–^-cotransporters include 4,4′-diisothiocyano-2,2′-stilbenedisulfonic acid (DIDS) [18] and S0859; [19–21] however, neither of these compounds show pharmacokinetic characteristics suited for in vivo administration or the pharmacological selectivity required for making solid inferences regarding molecular mechanisms.

The treatment of early breast cancer is in most cases a combination of surgery, radiation, systemic chemotherapy and/or endocrine agents. In disseminated breast cancer, treatment primarily relies on systemic therapy. Interfering with net acid extrusion from solid tumors is a promising treatment approach [22] but lack of specific pharmacological compounds has restricted preclinical development. Previous experimental interventions—in addition to genetic interference with acid-base targets in rodents—include ingestion of weak base (e.g., HCO_3_^–^ or TRIS) [23, 24] or combined intravital enzymatic release of weak acid and base [25] to increase buffering power at the tumor site and attenuate microenvironment acidity. However, oral dosing with NaHCO_3_ carry substantial, mainly gastrointestinal, adverse effects that reduce patient compliance and have resulted in clinical trial discontinuation [26]. Targeting acid-base transport proteins or downstream signaling pathways responding to tumor acidity could better tailor treatment to the cancer tissue and be less harmful to normal tissue.

NBCn1 shows several attractive characteristics as breast cancer therapeutic target. The cell surface expression makes NBCn1 readily available for extracellular binding; and the up to 7-fold upregulation of NBCn1 protein expression from normal breast tissue across pre-malignant tissue to full-blown cancer tissue supports therapeutic specificity [27]. Although NBCn1 has a broad expression profile in normal tissue [28], most non-cancerous cells experience a near-normal pH_o_ and a moderate metabolic acid load that they can adequately eliminate even if NBCn1 is inhibited. Accordingly, global constitutive NBCn1 knockout mice show only modest phenotypes until they are challenged, for instance, by aging, augmented acid-loading, substance abuse, carcinogen treatment, or infusion with vasoactive agonists [28–33]. The multiple splice variants of *SLC4A7*, with distinct tissue-specific expression profiles [34, 35], may permit predominant targeting of NBCn1 in disease-affected tissue. However, the known variable splice domains are restricted to the cytosolic compartment [34] and, therefore, not directly accessible for membrane-impermeable drugs, including traditional functional antibodies. Provided that anti-NBCn1 therapeutics—alone or in combination with other anti-cancer medications—are excluded from the central nervous system and administered for limited duration, we anticipate a mild and acceptable adverse drug reaction profile.

In this study, we tested the hypotheses that (a) functional antibodies targeting NBCn1 can be developed for specific pharmacological inhibition and (b) NBCn1 is responsible for the Na^+^,HCO_3_^–^-cotransport in human breast cancer tissue and can be targeted for breast cancer therapy.

## Materials and methods

**Fig. 1:**
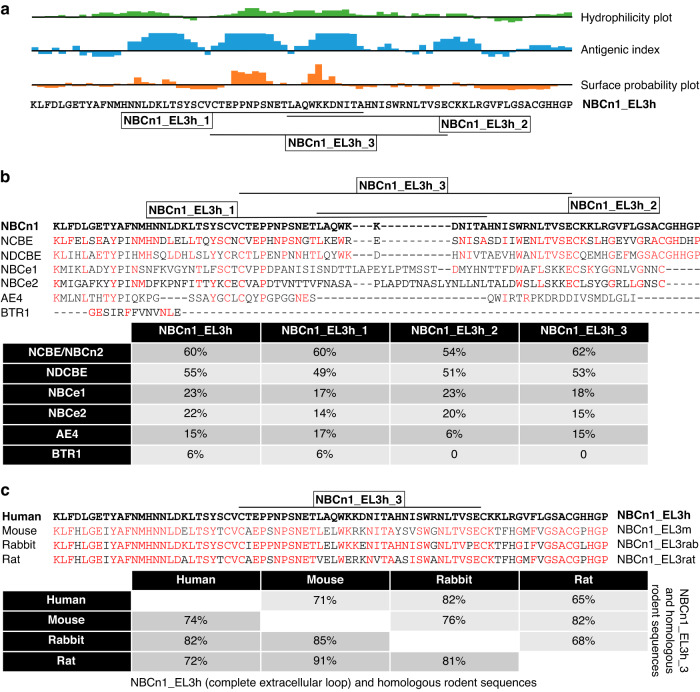
Epitopes within the third extracellular loop of NBCn1 selected for immunization, their sequence homologies to other Na^+^,HCO_3_^–^-cotransporters of the SLC4 family, and the degree of conservation between humans and rodents. **a** Hydrophilicity plot, antigenic index, and surface probability plot calculated for the third extracellular loop of human NBCn1. The annotations show the positions of the three peptides (NBCn1_EL3h_1-3) selected for immunization. **b** Sequence homologies between human NBCn1 and other known or putative Na^+^,HCO_3_^–^-cotransporters of the SLC4 family across the third extracellular loop (NBCn1_EL3h) and in the regions corresponding to the three selected peptides (NBCn1_EL3h_1-3). **c** Sequence homologies between human and rodent (mouse, rabbit, rat) NBCn1 across the third extracellular loop (NBCn1_EL3h) and in the region of the peptide (NBCn1_EL3h_3) used for monoclonal antibody development. **b**, **c** conserved amino acids relative to human NBCn1 are highlighted in red.

Building on two previous studies showing inhibitory actions of antibodies directed towards the third extracellular loop of NBCe1 (Slc4a4) [36, 37], we initially identified the corresponding regions of NBCn1. We based the further selection of specific peptide sequences for immunization on calculated biophysical properties and antigenic determinants within the predicted third extracellular loop of NBCn1 (GenScript, NJ, USA; Fig. 1a). The sequence homology between NBCn1 and the other known and putative Na^+^,HCO_3_^–^-cotransporters of the SLC4 family is less than 62% in the targeted region (Fig. 1b), and the degree of conservation is 65–91% between human, mouse, rabbit, and rat NBCn1 (Fig. 1c).

### Polyclonal antibody development

21^st^ Century Biochemicals (MA, USA) synthesized two partially overlapping hydrazine (Hz)-labeled peptides that they used for polyclonal antibody development: NBCn1_EL3h_1: [Hz]-HNNLDKLTSYSCVCTEPPNPSNETLAQWKKDNITA-amide and NBCn1_EL3h_2: [Hz]-LAQWKKDNITAHNISWRNLTVSECKKLRGVFLGSA-amide (Fig. 1). After keyhole limpet hemocyanin (KLH) conjugation, each peptide in Complete Freund’s Adjuvant was injected into two specific pathogen-free New Zealand rabbits that were subsequently boosted through injections of the same KLH-conjugated peptide in Incomplete Freund’s Adjuvant after 2, 4, 6, and 8 weeks. Serum was collected from blood sampled before the first immunization (pre-immune serum) and at week 7, 8, 10, and 11.

Based on enzyme-linked immunosorbent assay (ELISA) analysis, the collected serum samples were tested for binding to the immunizing peptides conjugated to bovine serum albumin (BSA). An anti-BSA antibody (#A11133, Life Technologies, Denmark) was used as positive control, and pre-immune serum served as negative control. Flat-bottomed 96-well cell culture plates (734–2327, VWR, Denmark) were coated overnight at 4 °C with BSA-conjugated peptide (diluted to 2 µg/mL in phosphate buffered saline (PBS); 60 µL per well) and then blocked with 15% fetal bovine serum (FBS; #S0115, Biochrom AG, Germany; 75 µL per well) in PBS for 1 h at 37 °C. Between each step, the plates were washed 4 times with 0.05% Tween-20 in PBS. Sera and the anti-BSA antibody were diluted in PBS, added to wells in duplicates (50 µL per well), and incubated for 1 h at 37 °C. After washing, the plates were incubated with horseradish peroxidase (HRP)-conjugated secondary goat anti-rabbit IgG (#70745, Cell Signaling Technology, MA, USA; 50 µL per well) for 1 h at 37 °C. After further washing, the plates were developed by incubation with the HRP substrate 3,3’,5,5’-tetramethylbenzidine (#002023, Life Technologies; 50 µL per well) for 30 min at 37 °C. Reactions were stopped by adding 100 µL of 1 M H_2_SO_4_ to each well. Absorbances were measured at 450 nm on a BioTek PowerWave 340 microplate spectrophotometer (Agilent, CA, USA) using 620 nm readings as reference.

**Fig. 2:**
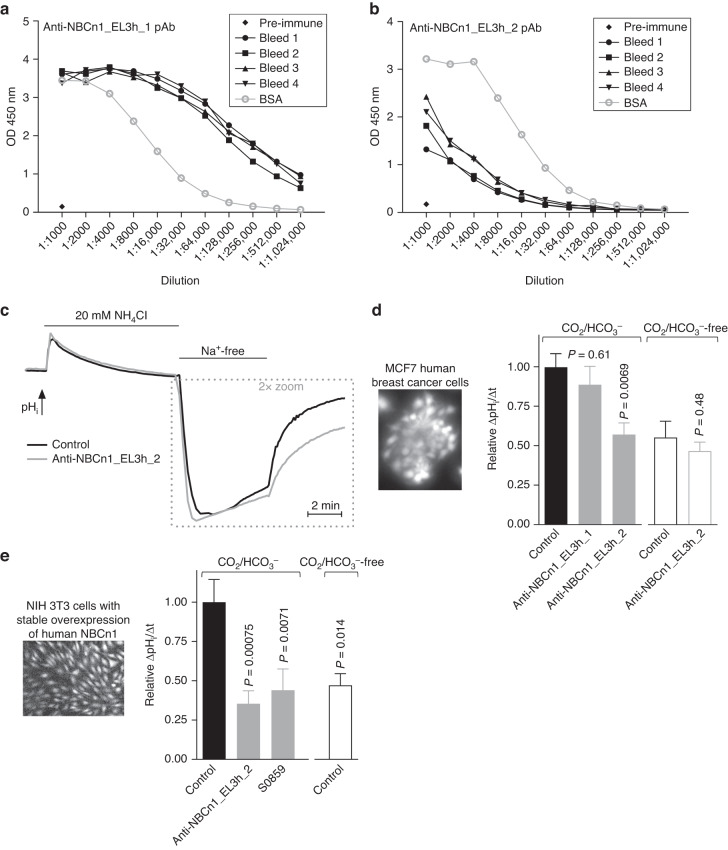
Rabbit polyclonal antibodies raised against the third extracellular loop of human NBCn1 inhibit Na^+^,HCO_3_^–^-cotransport activity in MCF7 human breast cancer cells and in NIH 3T3 cells with heterologous overexpression of human NBCn1. **a** + **b** ELISA-based analyses of binding between sera from rabbits injected with NBCn1_EL3h_1 (**a**) or NBCn1_EL3h_2 (**b**) and the corresponding bovine serum albumin (BSA)-conjugated peptides. **c** + **d** Average traces (**c**) and quantified pH_i_ recovery rates in presence of Na^+^ (**d**) show the effects of the two affinity purified polyclonal antibodies (15 µg/mL) on MCF7 cells during NH_4_^+^-prepulse-induced intracellular acidification (*n* = 9–20). Experiments were performed with or without CO_2_/HCO_3_^–^, as indicated. **e** Quantified pH_i_ recovery rates in presence of Na^+^ during NH_4_^+^-prepulse-induced intracellular acidification of NIH 3T3 cells with stable heterologous overexpression of human NBCn1 (*n* = 12–23). The bar chart shows effects of the purified polyclonal antibody (15 µg/mL) raised against NBCn1_EL3h_2, 30 µM of the small molecule inhibitor S0859 (supramaximal concentration of non-selective Na^+^,HCO_3_^–^-cotransport inhibitor), and omission of CO_2_/HCO_3_^–^, as indicated. We compared data by unpaired two-tailed Student’s *t*-test or one-way ANOVA followed by Dunnett’s post-test. Reported *P*-values relate to comparisons *vs*. control in similar buffer (**d**) or in presence of CO_2_/HCO_3_^–^ (**e**). OD optical density, pAb polyclonal antibody.

Sera with the highest titers were selected for affinity purification: we used pools of all four bleeds collected from rabbits injected with NBCn1_EL3h_1 (Fig. 2a) and pools of the last two bleeds collected from rabbits injected with NBCn1_EL3h_2 (Fig. 2b). Affinity purification was performed by 21^st^ Century Biochemicals using the synthetic peptides immobilized on optimized affinity resin. The purified antibodies were delivered as lyophilized powder that we stored at –20 °C. Stock solutions were prepared at concentrations of 1.5 mg/mL.

### Monoclonal antibody development

Peptide synthesis and development of monoclonal antibodies were performed by GenScript. The NBCn1_EL3h_3 peptide CTEPPNPSNETLAQWKKDNITAHNISWRNLTVSE-amide (Fig. 1) was KLH-conjugated and injected into five Balb/c and five C57Bl/6 mice. We identified the two mice showing the highest titers based on ELISA-tests of serum collected after the third immunization. The ELISA procedure was similar to that described above for polyclonal antibodies, except we used HRP-conjugated horse anti-mouse secondary antibody (#7076; Cell Signaling Technology) for detection. From the two selected mice, spleen cells were isolated and fused with SP2/0 mouse myeloma cells, and we screened supernatants from the resulting hybridoma cells by ELISA using the NBCn1_EL3h_3 peptide as coating antigen. The six primary hybridoma cell lines with highest titers were subcloned by limiting dilution; and from each cell line, two subclones were selected and expanded.

Using Protein G columns or beads, we affinity purified monoclonal antibodies from the supernatants of each of the 12 hybridoma subclones grown in DMEM, high glucose, pyruvate (#11995065; Thermo Fisher Scientific, Denmark) added 10% FBS, 1% penicillin/streptomycin, and 1% Glutamax. Larger quantities of monoclonal antibodies were generated by recombinant synthesis (Sino Biological, China). Hybridoma cells for selected antibodies underwent variable domain sequencing (GenScript), and the cDNA corresponding to the top candidate (5H2.1) was cloned into plasmids providing IgG1 isotype and mouse κ light chain backbone. Recombinant antibodies, transiently expressed in HEK293 cells, were purified by Protein A affinity chromatography (Sino Biological). The final antibody was tested for endotoxin contamination (<0.90 EU/mg), and purity was confirmed by sodium dodecyl sulfate-polyacrylamide gel electrophoresis (SDS-PAGE; 97.9%) and size exclusion high-performance liquid chromatography (SEC-HPLC; 99.9%). Antibodies were stored short-term at –20 °C and long-term at –80 °C.

### Analysis of cross-reactivity

In addition to the aforementioned three peptides from human NBCn1 (NBCn1_EL3h_1-3), we evaluated cross-reactivity using homologous peptides from human NBCe1 (NBCe1_EL3h_3: CVPPDPANISISNDTTLAPEYLPTMSSTDMYHN-amide) and murine NBCn1 (NBCn1_EL3m_3: CAEPSNPSNETLELWKRKNITAYSVSWGNLTVSE-amide). We also evaluated binding to a peptide corresponding to the fourth extracellular loop of human NBCn1 (NBCn1_EL4h_1): CPSPKLHVPEKFEPTHPERGWIISPLGDNPW-amide.

### Biacore affinity assays

We tested the binding affinity between the generated monoclonal antibodies and synthetic peptide antigens through surface plasmon resonance (SPR) on a Biacore T200 system (Cytiva, Denmark). The evaluated antibodies were captured on three of the flow cells on the SPR sensor chip (XanTec Bioanalytics GmbH, Germany) at a flow rate of 5 mL/min for 3 min, while the last flow cell acted as reference. Peptides were then injected at a flow rate of 5 mL/min to study association for 3 min and dissociation for 20 min. For kinetic analyses, the NBCn1_EL3h_3 peptide was applied at concentrations of 500, 250, 125, 62, 31, and 16 nM. To measure relative binding of different peptides, we applied 1 mM of the NBCn1_EL3h_3, NBCn1_EL4h_1, NBCe1_EL3h_3, or NBCn1_EL3m_3 peptides. All dilutions were performed using PBS with 10% dextran. Between each peptide or peptide concentration, the flow cell was regenerated using 10 mM glycine hydrochloride at pH 1.5 applied at a flow rate of 30 mL/min for 30 sec followed by a 3-min stabilization period. Experiments were performed at 25 °C and data collected at 1 Hz.

The data were fitted using the 1:1 interaction model of the Biacore T200 Evaluation Software to estimate association and dissociation rate constants and calculate the equilibrium dissociation constant (K_D_) as a measure of binding affinity. If the dissociation rate constant approached the instrument sensitivity, due to very slow dissociation kinetics, we also calculated the affinity limit as the ratio between the measured association rate constant and the specified instrument limit (1·10^–5^ s^–1^) of the dissociation rate constant.

### Cell lines

We used two human breast cancer cell lines: MCF7 cells of luminal A molecular subtype are positive for estrogen and progesterone receptors and have low metastatic potential [38], whereas CAL51 cells are triple-negative and derived from a pleural effusion in a patient with metastatic breast cancer [39, 40]. We purchased MCF7 cells from the American Type Culture Collection (ATCC, LGC Standards, UK). CAL51 cells were a kind gift from Dr. Issam Ben-Sahra (Northwestern University, USA). Both cell lines were grown in RPMI-1640 medium (#61870-010, Gibco, Denmark) supplemented with 6–10% FBS (#S0115, Biochrom AG) and 1% penicillin/streptomycin (#15140-122, Gibco). Cells were maintained at 37 °C in a humidified atmosphere of 5% CO_2_. MCF7 cells were tested regularly for mycoplasma contamination (GATC Biotech, Eurofins Scientific, Germany), seeded at passages 15–21 on cover glasses coated for 2 h with 0.2% autoclaved gelatine (#G2625, Sigma-Aldrich, Denmark), and investigated for intracellular pH (pH_i_) regulation after 2–3 days as described below.

NIH 3T3 mouse embryonic fibroblasts (Sigma-Aldrich) were grown in DMEM (Thermo Fisher Scientific) added 10% FBS, 1% penicillin/streptomycin (#15140-122, Gibco), and 1% Glutamax. Stable overexpression of human NBCn1 was achieved using liposome-based transfection with a pcDNA3.1^+^ plasmid containing a Cassette II-deficient variant of NBCn1 (mRNA Sequence ID: NM_001258380.1): [41] We mixed 1 mL DMEM, 5 µg plasmid DNA, and 15 µL XtremeGENE reagent (#06366244001; Roche, Denmark) for 15 min at room temperature to allow for lipid complex formation. Untransfected NIH 3T3 cells were grown to 50–60% confluency before incubation with the transfection mixture for 2 days. The DNA constructs underwent Sanger sequencing (Eurofins Scientific, Denmark). Transfected cells were grown in the presence of 500 µg/mL G418 (#G8168, Sigma-Aldrich) to eliminate sensitive parental cells and select for stably transfected cells. We further selected clones with prominent NBCn1 activity based on pH_i_ recordings (see below) demonstrating high capacity for Na^+^-dependent net acid extrusion. The NIH 3T3 cells were tested regularly for mycoplasma contamination.

### Human breast cancer tissue and isolation of primary organoids

With written informed consent, we sampled biopsies of fresh human primary breast cancer tissue from 25 women undergoing breast-conserving surgery at Aarhus University Hospital or Randers Regional Hospital, Denmark. Supplementary Table S1 summarizes the clinical and pathological patient and tumor characteristics. Data from a subset of the control group were reported as part of a previous study [17]. We included women, who were at least 18 years of age and presented with operable primary breast cancer (>10 mm) diagnosed by triple test including medical history, clinical examination, and mammography and ultrasonography of the breast combined with core-needle biopsy. None of the included patients had received pre-operative radiation or chemotherapy. We extracted information of the routine diagnostic procedures regarding patient age, tumor size, histology, malignancy grade, expression of estrogen receptors, HER2 status, Ki67 index, and lymph node metastasis from the medical records of the standard diagnostic care.

Progesterone receptor expression of breast cancer is not routinely evaluated in Danish clinical practice. Previous studies show that estrogen receptor negative breast cancer is generally also progesterone receptor negative [42] and that the rare cases of single progesterone receptor positive (i.e., ER^–^/PR^+^/HER2^–^) breast cancer show similar biological behavior and prognosis as triple-negative breast cancer [43]. Hence, we defined breast cancer as triple-negative if it showed no overexpression or gene amplification of HER2 and less than 10% of cells were positive for estrogen receptors.

We isolated primary epithelial organoids from breast cancer tissue as previously described [3, 9, 10, 27, 44]. The breast tissue was first mechanically disrupted into small pieces with scalpels and then transferred to T25 culture flasks containing 89% advanced DMEM/F12 culture medium (Thermo Fisher Scientific), 10% fetal bovine serum (Biochrom AG), 1% Glutamax (Thermo Fisher Scientific), and a final concentration of 450 IU/mL collagenase type III (Worthington Biochemical Corporation, NJ, USA). After overnight incubation at 37 °C in a humidified atmosphere of 5% CO_2_, a small volume of the solution was transferred to an Eppendorf tube, and organoids were left to sediment by gravitational forces. The primary organoids, freshly isolated by this technique, consist predominantly of cytokeratin-19 positive epithelial cells with few smooth muscle α-actin positive myofibroblast [3, 9], and we used them directly for pH_i_ recordings without culture.

### Intracellular pH recordings

MCF7 cells, NIH 3T3 cells with stable overexpression of human NBCn1, and primary organoids freshly isolated from human breast cancer tissue were loaded with 3 µM BCECF-AM (#B-1170, Invitrogen, Thermo Fisher Scientific) for 20–30 min at 37 °C. The preparations were mounted in a custom-built chamber [45]—continuously heated to 37 °C and aerated with 5% CO_2_/balance air or nominally CO_2_-free air—on the stage of an Olympus IX70 or Nikon Diaphot 200 microscope. BCECF-loaded cells and primary organoids were excited alternatingly at 490 nm and 440 nm and emission light collected at 510 nm with charge-coupled device-based fluorescence imaging systems controlled through EasyRatioPro (Photon Technology International, NJ, USA) or VisiView (Visitron Systems, Germany) software.

We induced intracellular acidification using the NH_4_^+^-prepulse technique: [46] 20 mM NH_4_Cl was added to the experimental bath solution for 15 min before it was washed out to a Na^+^-free solution in order to first evaluate the rate of Na^+^-independent net acid extrusion. Then, Na^+^ was returned to the bath solution to activate Na^+^-dependent net acid extrusion mechanisms (Fig. 2c). We also investigated regulation of pH_i_ in absence of CO_2_/HCO_3_^–^ to distinguish Na^+^,HCO_3_^–^-cotransport from Na^+^/H^+^-exchange. In cell lines, we analyzed the rate of BCECF fluorescence ratio change during the first 30 s after readdition of Na^+^; and we report this measure—which is proportional to the pH_i_ recovery rate (ΔpH_i_/Δt)—relative to control conditions without antibody added. In breast cancer tissue, we calibrated the ratiometric BCECF signal to pH using the high-[K^+^] nigericin technique; and we then calculated Na^+^-dependent net acid extrusion at pH_i_ 6.5 taking into account the intracellular buffering capacity and assuming a linear transporter activation profile with decreasing pH_i_ [17]. We analyzed resting pH_i_ as the steady-state pH_i_ after recovery from NH_4_^+^-prepulse-induced acidification.

We estimated the intrinsic buffering capacity from the change in pH_i_ upon addition and washout of NH_4_Cl in absence of CO_2_/HCO_3_^–^. The contribution of CO_2_/HCO_3_^–^ to intracellular buffering capacity was calculated as 2.3 × [HCO_3_^–^]_i_ [47]. Concentrations of NH_4_^+^ and HCO_3_^–^ were calculated based on the Henderson-Hasselbalch equation. For all included patients, we obtained matched data on pH_i_ regulation with and without antibody and in the presence and absence of CO_2_/HCO_3_^–^. A few organoids were excluded because the initial pH_i_ value after loading with BCECF varied more than 0.5 between matched samples.

The physiological saline solution used for pH_i_ recordings contained (in mM): [48] 140 Na^+^, 4 K^+^, 1.6 Ca^2+^, 1.2 Mg^2+^, 124 Cl^−^, 22 HCO_3_^−^, 1.2 SO_4_^2−^, 1.18 H_2_PO_4_^−^, 10 HEPES, 5.5 glucose, 0.03 EDTA. In CO_2_/HCO_3_^–^-free solutions, HCO_3_^–^ was replaced with equimolar Cl^–^. In Na^+^-free solutions, Na^+^ was replaced with equimolar *N*-methyl-D-glucammonium; except for NaHCO_3_, which was replaced with choline-HCO_3_. CO_2_/HCO_3_^–^-containing solutions were aerated with 5% CO_2_/balance air, whereas CO_2_/HCO_3_^–^-free solutions were bubbled with nominally CO_2_-free air. All solutions were adjusted to pH 7.4 at 37 °C. Solutions contained 5 mM probenecid to inhibit BCECF extrusion by the organic anion transporter. The affinity purified anti-NBCn1 antibodies were solubilized in distilled water (1–1.5 mg/mL) and added to the experimental solutions at 1:100 dilution from the time of NH_4_Cl addition until the end of the experiment.

### Immunoblotting and surface biotinylation

MCF7 cells were cultivated in RPMI-1640 medium added 20 nM 5H2.1 antibody or a corresponding volume of PBS vehicle at pH 7.4 for 1 h in an incubator maintained at 37 °C and 5% CO_2_. Cells were washed twice and scraped in ice-cold PBS, centrifuged at 250 *g*, and resuspended and incubated for 30 min in 400 µL PBS (pH 8.4) mixed with 100 µL EZ-Link™ NHS-LC-LC-Biotin (#21343, Thermo Fisher Scientific; 10 mM in pure water) under gentle rotation at 4 °C. The biotinylation reaction was quenched by addition of 100 mM glycine in PBS, added first to the reaction tubes for 5 min under gentle rotation and then, after centrifugation, through a triple-wash. Protein concentrations were determined with a bicinchoninic acid protein assay kit (#23227, Thermo Fisher Scientific); and 1 mg protein diluted in 400 µL lysis buffer containing HALT Protease Inhibitor Cocktail (#78430, Thermo Fisher Scientific) was added to BSA-blocked Pierce Monomeric Avidin Beads (#20228, Thermo Fisher Scientific) and kept under rotation for 4 h at 4 °C to bind the biotinylated proteins. Unlabeled lysates were used as negative control. The beads were washed three times with PBS containing 0.1% Tween-20 and HALT Protease Inhibitor Cocktail, and the bound proteins were eluted in 45 µL of 4 × laemmli buffer (#1610747, BIO-RAD, Denmark) by heating to 95 °C with regular vortexing. After centrifugation, 5% dithiothreitol was added to the samples. We next loaded 20 µL of the eluted biotinylated protein fraction or 10 µg of total protein from whole-cell lysates in each lane of a stain-free sodium dodecyl sulfate polyacrylamide gel (BIO-RAD) for electrophoresis, total protein determination on an Azure C600 Imaging System (Azure Biosystems, CA, USA), and subsequent transfer to polyvinylidene difluoride membranes blocked with 5% skimmed milk. The membranes were probed with rabbit anti-NH_2_-terminal NBCn1 antibody (kind gift from Dr. Jeppe Praetorius, Aarhus University) that we had affinity purified using the immunizing peptide (21st Century Biochemicals) [49]. After thorough washing, membranes were incubated with secondary goat anti-rabbit antibody conjugated to HRP (#7074, Cell Signaling Technology). Bound antibody was detected by enhanced chemiluminescence (ECL Plus; GE Healthcare, Denmark) using an Azure C600 Imaging System. We quantified band intensities relative to the loading control using ImageJ software (Rasband, National Institutes of Health, USA).

### Patient-derived xenograft models

To test the in vivo implications of the developed anti-NBCn1 inhibitory antibodies, we conducted studies on patient-derived xenograft models. The experiments were performed commercially by Charles River Laboratories (Germany). A research protocol outlining the experimental procedures was prepared with Charles River before study initiation.

First, a tissue microarray consisting of tumor tissue samples excised from tumor‐bearing nude mice was immunohistochemically stained to evaluate NBCn1 expression in available patient-derived xenograft models. Tumor material was fixed in 10% neutral-buffered formalin for approximately 24 h and then placed in 70% (V/V) ethanol for 1–7 days. Subsequent dehydration and paraffin embedding was carried out through graded washes to 70% ethanol (1 h), 80% ethanol (2 h), 99% ethanol (1 h), two times 100% isopropanol (0.5 h; 1 h), three times xylene (0.5 h; 1 h; 1 h), and three times paraffin (1 h; 2 h; 2 h). Then, paraffin-embedded tumors were cut to 5 µm thick sections, dried and dewaxed, and stained with hematoxylin and eosin. These “whole tumor” sections were inspected; and from a representative region, a 1-mm cone was punched out for inclusion in the tissue microarray. Antigen retrieval was performed for 5 min in Tris-EDTA buffer (pH 9) with a pressure cooker (level Lo1). Slides at room temperature were permeabilized, treated with 3% H_2_O_2_ and Dako Protein Block, each for 5 min, and incubated with 6.13 µg/mL primary anti-*N*-terminal NBCn1 antibody [49] in Dako antibody diluent for 60 min. Corresponding isotype controls showed no staining.

Next, we evaluated the functional impact of the anti-NBCn1 inhibitory antibodies on tumor growth in two selected patient-derived xenograft models of triple-negative breast cancer with prominent NBCn1 expression. Female NMRI^nu/nu^ mice were engrafted subcutaneously with MAXFTN BR120 tumor material (at passage 9–12) from a 52-year old female with an undifferentiated breast cancer showing 5% stroma content and low vascularization, or with MAXFTN 2988 (at passage 9–11) from a 42-year old female with a high-grade breast adenocarcinoma showing moderate differentiation. On day 17–26 after tumor implantation, the tumor-bearing mice were randomized to anti-NBCn1 antibody treatment or PBS vehicle. Sample sizes were selected to identify tumor growth influences, confounders controlled, and randomization and blinding performed based on the prior experience of Charles River Laboratories. Treatment with twice weekly intraperitoneal injections of 200 mg/kg 5H2.1 antibody or equivalent volume (10 mL/kg) of PBS vehicle continued for up to 6 weeks. Tumor sizes and body weights were measured twice weekly. The tumors that were smallest (initial volume < 100 mm^3^) at the time of randomization showed unpredictable growth patterns with large standard deviations of tumor volume measurements after 21 days compared to the initially larger tumors (initial volume > 100 mm^3^). We therefore limited our analyses to tumors > 100 mm^3^ at the time of randomization and treatment initiation. The treatment was well tolerated, and we show the exponential tumor growth phase up until the day when the first mouse in the group passed the ethically permitted tumor load of 2000 mm^3^.

### In vitro analyses of lactate, colony formation, cell proliferation and viability, cell cycle, and cell death

We assessed CAL51 cells cultivated in RPMI-1640 culture medium that was adjusted to pH 7.4 or 6.8 by addition of HCl at 37 °C in a humidified atmosphere of 5% CO_2_. The pH levels were chosen to mimic physiological conditions and the tumor microenvironment, respectively.

To evaluate glycolytic activity, we seeded CAL51 cells in 24-well plates (#142485, Thermo Fisher Scientific) at a density of 100,000 cells/well. We collected media samples after 24 h of cell culture in RPMI-1640 medium added 20 nM 5H2.1 antibody or equivalent volume of PBS vehicle. We measured lactate concentrations in the media samples using an ISCUSflex Analyzer (M Dialysis, Sweden) and subtracted the signal obtained from similar medium that had not been used for cell culture.

We examined colony formation after seeding CAL51 cells in 6-well plates (#140675, Thermo Fisher Scientific) at a density of 500 cells/well. For 3 weeks, the cells were grown in RPMI-1640 medium added 20 nM 5H2.1 antibody or equivalent volume of PBS vehicle. At the end of the treatment period, cells were stained with methylene blue for 1 h, and the colonies were counted using ImageJ software.

We estimated numbers of viable cells using the colorimetric 3‐(4,5‐dimethylthiazol‐2‐yl)‐2,5‐diphenyltetrazolium bromide (MTT) assay (#M6494, Thermo Fisher Scientific). CAL51 cells were seeded in 96-well plates (#260887, Thermo Fisher Scientific) at a density of 15,000 cells/well. The cells were grown for 3 days in RPMI-1640 medium added 20 nM 5H2.1 antibody or equivalent volume of PBS vehicle. Next, the cells were washed to 200 µL fresh RPMI-1640 medium containing MTT solution (2 mg/mL). After 4 h, the supernatants were aspirated, and 200 µL DMSO was added to each well followed by 30 min of incubation at 37 °C. Absorbance was measured at 570 nm using a BioTek PowerWave 340 microplate reader.

We performed cell cycle assays based on propidium iodide staining of DNA. CAL51 cells were seeded in 24-well plates (#142485, Thermo Fisher Scientific) at a density of 100,000 cells/well. The cells were grown in RPMI-1640 medium added 20 nM 5H2.1 antibody or equivalent volume of PBS vehicle for 24, 48 or 72 h. At the indicated time points, cells were harvested using trypsin (#15090-046, Thermo Fisher Scientific), washed and resuspended in 500 µL cold PBS, and then fixed by addition of 4 mL cold 75% ethanol followed by vortexing and incubation for at least 2 h. The cells were then washed with cold PBS and treated with 50 µL DNase- and protease-free RNase A (100 µg/mL; #EN0531, Thermo Fisher Scientific) for 30 min at room temperature. Finally, the cells were stained with 100 µL propidium iodide (20 µg/mL) for 30 min at 4 °C and analyzed on a NovoCyte Quanteon flow cytometer (Agilent) using the 488 nm laser and 575 nm detection. For data analysis, we used NovoExpress (v. 1.6.2, Agilent) and FlowJo (v10.8.1, BD Biosciences, Denmark) software.

We evaluated cell death based on the Invitrogen Dead Cell Apoptosis Kit (#V13245, Thermo Fisher Scientific) according to the manufacturer’s instructions. Briefly, CAL51 cells were seeded in 24-well plates (#142485, Thermo Fisher Scientific) at a density of 100,000 cells/well. The cells were grown in RPMI-1640 medium added 20 nM 5H2.1 antibody or equivalent volume of PBS vehicle for 12, 24, 48 or 72 h. At the indicated time points, cells were harvested using trypsin (#15090-046, Thermo Fisher Scientific) and washed in cold PBS. The cells were then resuspended in 1 × Annexin Binding Buffer and stained with propidium iodide and Alexa Fluor^®^ 488-labeled Annexin V for 20 min at room temperature in the dark. Finally, the cells were analyzed on a NovoCyte Quanteon flow cytometer using the 488 nm laser and detection at 530 and 575 nm. Based on the Annexin V (AV) and propidium iodide (PI) signals, we used NovoExpress and FlowJo software to divide between viable (AV^–^/PI^–^), necrotic (AV^–^/PI^+^), early apoptotic (AV^+^/PI^–^), and late apoptotic (AV^+^/PI^+^) cells.

### Statistics

Data are expressed as mean ± SEM. The n-values are stated in the figure legends and report the number of experiments, wells, mice, or patients. Distributions were evaluated visually for normality using Q-Q plots. In case of right-skewed data or unequal variance between groups, we performed logarithmic transformation before comparisons. Sample sizes were chosen based on prior experience. A single distribution was compared to a hypothetical value using one-sample *t*-test. Single variables were compared between two groups by unpaired, two-tailed Student’s *t*-test or between more than two groups by one-way ANOVA followed by Dunnett’s post-test. We evaluated the influence of two independent variables on a third variable by ordinary or repeated measures two-way ANOVA followed by Šidák’s post-test. Tumor growth curves were fitted to one-phase exponential functions and compared by extra sum-of-squares *F*-tests. *P* < 0.05 was considered statistically significant. Statistical analyses were performed using GraphPad Prism 10.0.0 software.

## Results

### Antigen design

We generated antibodies against the third extracellular loop of NBCn1 by injecting rabbits and mice with immunizing peptides 34–35 amino acids in length (Fig. 1). We based the peptide design on previous reports [36, 37] regarding NBCe1 and analyses of antigenicity and extracellular accessibility (Fig. 1a). The targeted regions within the third extracellular loop of NBCn1 showed up to 62% identity with the other known or putative Na^+^,HCO_3_^–^-cotransporters of the SLC4 family (Fig. 1b) and were 65–91% conserved between human and rodent (mouse, rabbit, rat) NBCn1 (Fig. 1c).

### Polyclonal antibodies against NBCn1 inhibit transport activity

Serum from rabbits injected with either the NBCn1_EL3h_1 or NBCn1_EL3h_2 peptides (Fig. 1) showed immunoreactivity against the corresponding peptides when assessed by ELISA (Fig. 2a, b).

Next, we evaluated the influence of antibodies affinity purified from the serum of the immunized rabbits on net acid extrusion from MCF7 human breast cancer cells during intracellular acidification (Fig. 2c). The titer was higher for rabbits injected with NBCn1_EL3h_1 compared to NBCn1_EL3h_2 (Fig. 2a, b); but when applied to MCF7 human breast cancer cells, the anti-NBCn1_EL3h_2 antibody inhibited literally all Na^+^,HCO_3_^–^-cotransport whereas the anti-EL3h_1 antibody showed no significant effect (Fig. 2d). The anti-NBCn1_EL3h_2 antibody had no effect on net acid extrusion in the absence of CO_2_/HCO_3_^–^ (Fig. 2d).

Based on siRNA-mediated knockdown, Na^+^,HCO_3_^–^-cotransport in MCF7 cells was previously found dependent on NBCn1 [50]. However, to confirm more directly the effect of the anti-NBCn1_EL3h_2 antibody on NBCn1, we next generated NIH 3T3 cells with heterologous overexpression of human NBCn1. In these cells, we found again that the anti-NBCn1_EL3h_2 antibody was able to block all Na^+^,HCO_3_^–^-cotransport (Fig. 2e). We further showed that the effect of the anti-NBCn1_EL3h_2 antibody was similar to that of the small molecule inhibitor S0859 (Fig. 2e) applied at a supramaximal concentration of 30 µM.

### Monoclonal antibodies against NBCn1 inhibit transport activity

**Fig. 3:**
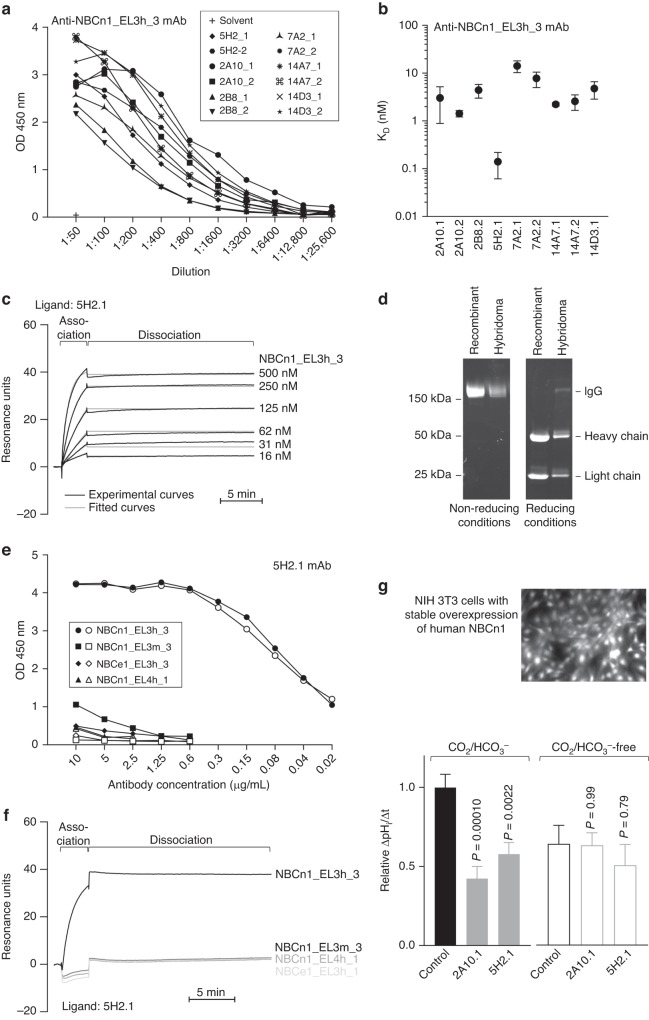
High-affinity mouse monoclonal antibodies raised against the third extracellular loop of NBCn1 inhibit Na^+^,HCO_3_^–^-cotransport in NIH 3T3 cells with stable heterologous overexpression of human NBCn1. **a** + **e** ELISA-based analysis evaluating the binding of purified mouse monoclonal antibodies to the NBCn1_EL3h_3 peptide used for immunization (**a**) and related peptides from NBCn1 and NBCe1 (**e**). Closed symbols correspond to hybridoma antibodies; open symbols in (**e**) correspond to recombinant antibodies. **b** + **c** Biacore-based surface plasmon resonance analyses. Binding affinities (**b**; *n* = 3–10) calculated from experiments (**c**) evaluating the interaction between purified mouse monoclonal antibodies and the NBCn1_EL3h_3 peptide used for immunization. The average antibody capture levels were 450 ± 49 resonance units for the hybridoma and 589 ± 74 resonance units for the recombinant 5H2.1 antibody. **d** Electrophoretically separated hybridoma and recombinant 5H2.1 antibodies under non-reducing and reducing conditions. **f** Biacore-based surface plasmon resonance analysis evaluating the binding of the purified 5H2.1 antibody to the NBCn1_EL3h_3 peptide used for immunization and related peptides from NBCn1 (NBCn1_EL4h_1, NBCn1_EL3m_3) and NBCe1 (NBCe1_EL3h_3) each applied at 1 mM. The antibody capture levels for the displayed traces were between 687 and 712 resonance units. **g** Quantified pH_i_ recovery rates in presence of Na^+^ during NH_4_^+^-prepulse-induced intracellular acidification of NIH 3T3 cells with stable heterologous overexpression of NBCn1 (*n* = 5–43). The bar chart shows effects of two purified monoclonal antibodies (10–33 nM 2A10.1 and 5H2.1) in experiments performed with or without CO_2_/HCO_3_^–^, as indicated. Data were compared by unpaired, two-tailed Student’s *t*-test or one-way ANOVA followed by Dunnett’s post-test. Reported *P*-values relate to comparisons *vs*. control in similar buffer. *K*_D_ equilibrium dissociation constant, mAb monoclonal antibody, OD optical density.

We next developed mouse monoclonal antibodies against the NBCn1_EL3h_3 peptide (Figs. 1 and 3). All 12 subclones of hybridoma cells generated produced antibodies reactive towards the NBCn1_EL3h_3 peptide based on ELISA (Fig. 3a).

We used Biacore assays to further evaluate the affinity of the developed monoclonal antibodies. As illustrated in Fig. 3b, c, the 5H2.1 antibody showed particularly high affinity (*K*_D_ = 0.14 nM) towards the third extracellular loop of human NBCn1 whereas the other antibodies had affinities that were 10–100-fold lower (*K*_D_ = 1.43–14.2 nM). Due to the very slow dissociation of the 5H2.1 antibody from the NBCn1_EL3h_3 peptide, the estimated dissociation rate constant in several experiments was outside the range specified for the Biacore equipment. Using the instrument limit, we can conclude that the equilibrium dissociation constant (*K*_D_) of 5H2.1 is ≤0.17 ± 0.02 nM.

We produced larger amounts of the 5H2.1 antibody by recombinant techniques (Fig. 3d). Binding to the NBCn1_EL3h_3 peptide was similar for the hybridoma and recombinant 5H2.1 antibodies and neither showed meaningful binding to murine NBCn1 (NBCn1_EL3m_3), human NBCe1 (NBCe1_EL3h_3), or regions of human NBCn1 (NBCn1_EL4h_1) outside the third extracellular loop based on ELISA (Fig. 3e) or Biacore analysis (Fig. 3f).

The high-affinity 5H2.1 antibody and the lower-affinity 2A10.1 antibody both inhibited all Na^+^,HCO_3_^–^-cotransport activity when applied at 10–33 nM concentration to NIH 3T3 cells overexpressing human NBCn1, and neither antibody influenced pH_i_ regulation in the absence of CO_2_/HCO_3_^–^ (Fig. 3g).

Incubation of MCF7 cells with the 5H2.1 antibody for 1 h had no effect on total (Supplementary Fig S1A, B) or cell surface (Supplementary Fig S1A, C) expression of NBCn1, supporting that the inhibitory effect—at least in the acute phase—is due to inactivation of the transporter, rather than internalization and degradation.

### NBCn1 mediates the Na^+^,HCO_3_^–^-cotransport in human breast cancer tissue

**Fig. 4:**
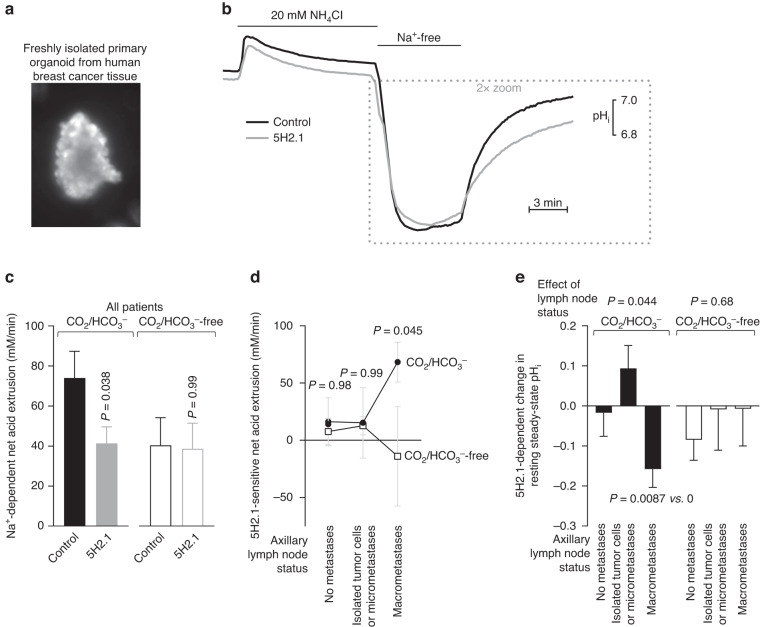
NBCn1-directed inhibitory antibodies block net acid extrusion from human breast cancer tissue, especially from patients with macrometastases in the axillary lymph nodes. **a** Fluorescence image of BCECF-loaded primary organoid freshly isolated from human breast cancer tissue. The diameter of organoids is 100–150 µm [3, 9]. **b** Average traces of pH_i_ during NH_4_^+^-prepulse-induced intracellular acidification of organoids isolated from primary breast carcinomas. The illustrated experiments were performed in the presence of CO_2_/HCO_3_^–^ on tissue from patients with macrometastases in the axillary lymph nodes. The 5H2.1 antibody was added at 10 nM from 30 min before addition of NH_4_Cl until the end of the experiment. **c** Na^+^-dependent net acid extrusion calculated at pH_i_ 6.5 under conditions with and without CO_2_/HCO_3_^–^, as indicated. Data are presented for the whole group of patients (*n* = 25). **d** + **e** 5H2.1-sensitive net acid extrusion calculated at pH_i_ 6.5 (**d**) and 5H2.1-dependent changes in steady-state pH_i_ (**e**) in the presence and absence of CO_2_/HCO_3_^–^ and stratified between patients without lymph node metastases (*n* = 12), with isolated tumor cells or micrometastases (*n* = 5), and with macrometastases (*n* = 8) in the axillary sentinel lymph nodes. Data in (**c**, **d**) were compared by two-way ANOVA followed by Šidák’s post-tests. Data in (**e**) were compared by one-way ANOVA and one-sample *t*-test. Reported *P*-values relate to comparisons *vs*. control in similar buffer (**c**) or matched recordings without CO_2_/HCO_3_^–^ (**d**).

Previous studies based on transgenic approaches in mice show that NBCn1 is responsible for the Na^+^,HCO_3_^–^-cotransport in murine breast cancer tissue. NBCn1 is expressed in the human breast epithelium and upregulated during breast carcinogenesis [3, 11, 17]. However, due to lack of specific pharmacological inhibitors, we have not previously had direct functional evidence for contribution from NBCn1 to net acid extrusion in human breast cancer tissue. Therefore, we next tested whether the 5H2.1 antibody inhibits Na^+^,HCO_3_^–^-cotransport in human breast cancer tissue (Fig. 4). We obtained fresh tissue biopsies directly from breast-conserving surgeries, generated primary organoids by acute partial digestion (Fig. 4a), and measured pH_i_ during NH_4_^+^-prepulse-induced intracellular acidification (Fig. 4b). The Na^+^-dependent net acid extrusion mediated by Na^+^,HCO_3_^–^-cotransport in the primary organoids was completely inhibited by the anti-NBCn1 5H2.1 monoclonal antibody (Fig. 4c). The 5H2.1 antibody did not affect net acid extrusion in the absence of CO_2_/HCO_3_^–^ (Fig. 4c). These data provide the first direct evidence that NBCn1 is exclusively responsible for the net acid extrusion mediated by Na^+^,HCO_3_^–^-cotransport in human breast cancer tissue under the evaluated conditions.

### NBCn1-mediated Na^+^,HCO_3_^–^-cotransport predicts human regional lymph node metastasis

We recently showed that NBCn1 expression and overall Na^+^,HCO_3_^–^-cotransport activity in breast cancer tissue independently predict regional lymph node metastasis in patients [17]. To substantiate the role of NBCn1-mediated Na^+^,HCO_3_^–^-cotransport, we therefore stratified the patient cohort from Fig. 4c into three groups based on the axillary sentinel lymph node status: the first group was without metastases, the second had lymph nodes containing isolated tumor cells (≤200 cells and ≤0.2 mm) or micrometastases (>200 cells or >0.2 mm, but <2 mm), and the third had lymph nodes containing macrometastases (≥2 mm). For each group, we then calculated the 5H2.1-sensitive net acid extrusion in the presence and absence of CO_2_/HCO_3_^–^. This additional evaluation shows that the 5H2.1-sensitive (i.e., NBCn1-mediated) Na^+^,HCO_3_^–^-cotransport is strongly elevated in patients with simultaneous macrometastases at time of diagnosis compared to patients without lymph node involvement (Fig. 4d). The higher NBCn1 activity in tumors from patients with axillary macrometastases was also reflected in a greater decrease in resting steady-state pH_i_ when organoids were treated with the 5H2.1 antibody in presence of CO_2_/HCO_3_^–^ (Fig. 4e).

### Anti-NBCn1 inhibitory antibodies can decelerate breast cancer growth

**Fig. 5:**
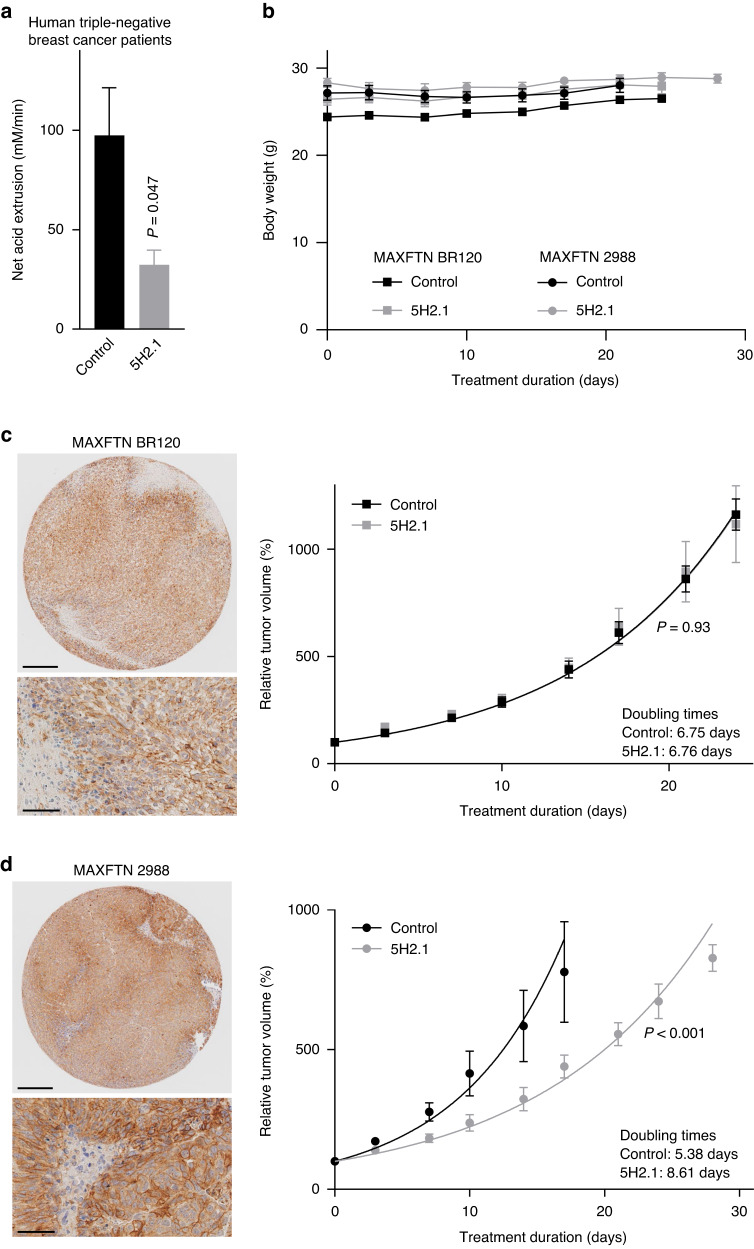
Proof-of-concept that NBCn1-directed inhibitory antibodies can decelerate breast tumor growth. **a** Net acid extrusion in organoids isolated from human breast carcinomas with normal HER2 expression and low estrogen receptor expression (*n* = 3). The data from the patients with this expression profile were extracted from the evaluated human cohort (Supplementary Table S1), are also included in Fig. 4c–e, and were compared by paired two-tailed Student’s *t*-test. **b** Weight development of mice engrafted with patient-derived xenograft models of triple-negative breast cancer during treatment with anti-NBCn1 5H2.1 antibody or PBS vehicle. **c** + **d** Immunohistochemical staining for NBCn1 and growth curves during treatment with anti-NBCn1 5H2.1 antibody or PBS vehicle (*n* = 5–6). The data are from patient-derived xenograft models of triple-negative breast cancer and include tumors that were >100 mm^3^ at treatment initiation. **c** 52-year old female, 5% stroma content, low vascularization, undifferentiated. **d** 42-year old female, high-grade adenocarcinoma, moderate differentiation. The data were fitted to exponential growth curves, and we compared the growth constants by extra sum-of-squares *F*-tests. Scale bars are 200 µm on low magnification images (upper panels) and 50 µm on high magnification images (lower panels). Reported *P*-values relate to comparisons *vs*. control.

Prior experimental evidence based on genetic disruption in mice [9, 10] or shRNA-mediated knockdown in xenografted cell lines [51] supports that NBCn1 accelerates breast cancer growth. To explore this effect further and substantiate the potential of acute pharmacological blockade, we used patient-derived xenograft models and treated the engrafted mice with the 5H2.1 antibody for up to 6 weeks (Fig. 5). Our previous studies found that *SLC4A7* mRNA expression levels predict survival specifically in patients with luminal A and basal-like/triple-negative breast cancer [17]. From our human cohort (Fig. 4c), we extracted data from patients with triple-negative breast cancer and found a dramatic 5H2.1-mediated inhibition of net acid extrusion (Fig. 5a). Based on these observations, we focused the in vivo experiments on models of triple-negative breast cancer.

From immunohistochemical stainings for NBCn1 (Fig. 5c, d), we first selected two patient-derived xenograft models of human triple-negative breast cancer. We observed membrane-localized protein expression of NBCn1 widely across the tumors; especially throughout the epithelial components but also at lower intensity in stromal areas (Fig. 5c, d).

Treating mice carrying tumors of the two selected patient-derived xenograft models with the 5H2.1 antibody revealed no clinical adverse effects, and the mice showed stable body weight throughout the treatment period (Fig. 5b). In one of the two selected models, the 5H2.1 antibody delayed tumor growth by around 50% (Fig. 5d). In the other model, we observed no effect of the 5H2.1 antibody relative to vehicle injection (Fig. 5c).

### NBCn1-directed inhibitory antibodies do not alter glycolytic activity

To test the cellular influences of the inhibitory anti-NBCn1 antibodies in greater detail, we next studied effects of the 5H2.1 antibody on the human CAL51 cell line, which is derived from a patient with metastatic triple-negative breast cancer [39, 40].

Fermentative glycolysis is pH sensitive in several settings [8], and we therefore first tested whether inhibition of NBCn1 influenced lactate production. We saw no effect of lowering pH_o_ from 7.4 to 6.8 or of adding 20 nM of the 5H2.1 antibody on lactate accumulation in the culture medium used for growing CAL51 cells (Supplementary Fig S2).

### NBCn1-directed inhibitory antibodies cause pH-dependent G2/M-phase cell cycle accumulation and increased apoptosis

**Fig. 6:**
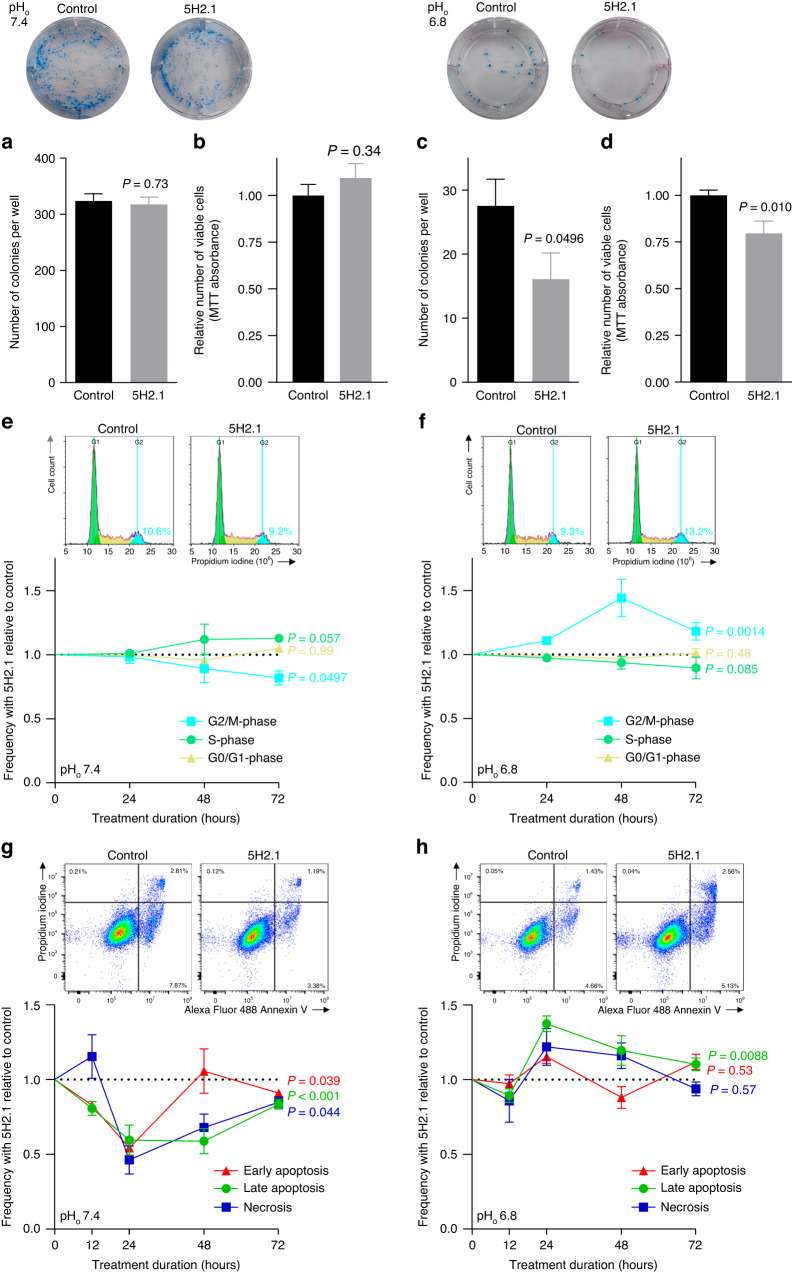
NBCn1-directed inhibitory antibodies limit expansion of CAL51 human metastatic triple-negative breast cancer cells, cause G2/M-phase cell cycle accumulation, and increase apoptosis under acidic conditions. **a** + **c** Colony formation after 3 weeks of culture at pH_o_ 7.4 (**a**, *n* = 9) or 6.8 (**c**, *n* = 9) in the presence of 20 nM 5H2.1 or control vehicle. **b** + **d** Relative numbers of viable cells after incubation with 20 nM 5H2.1 or control vehicle for 72 h at pH_o_ 7.4 (**b**, *n* = 9–15) or 6.8 (**d**, *n* = 15). **e** + **f** Cell cycle analyses showing exemplar histograms and quantified effects of 20 nM 5H2.1 antibody compared to control vehicle over the first 72 h of treatment at pH_o_ 7.4 (**e**, *n* = 6) or 6.8 (**f**, *n* = 6). In the control group at pH_o_ 7.4, 54.5 ± 0.7% of cells were in G0/G1 phase, 28.0 ± 1.0% in S phase, and 14.8 ± 1.1% in G2/M phase. In the control group at pH_o_ 6.8, 54.5 ± 0.6% of cells were in G0/G1 phase, 29.4 ± 0.8% in S phase, and 12.4 ± 0.8% in G2/M phase. The exemplar histograms are from 48 h incubation; the numbers on the histograms indicate the percentage of cells in G2/M phase. **g** + **h** Exemplar flow cytometry bivariate density plots and quantification of apoptosis and necrosis during incubation with 20 nM 5H2.1 or control vehicle over the first 72 h of treatment at pH_o_ 7.4 (**g**, *n* = 5–6) or 6.8 (**h**, *n* = 5–6). In the control group at pH_o_ 7.4, 92.77 ± 0.81% of cells were viable, 0.82 ± 0.28% necrotic, 3.99 ± 0.56% early apoptotic, and 2.41 ± 0.30% late apoptotic. In the control group at pH_o_ 6.8, 94.24 ± 0.53% of cells were viable, 0.51 ± 0.18% necrotic, 3.27 ± 0.31% early apoptotic, and 1.97 ± 0.24% late apoptotic. The representative flow cytometry images are from 24 h incubation; the numbers on the density plots indicate the percentage of cells in that quadrant. Data in (**a**–**d**) were compared by unpaired two-tailed Student’s *t*-tests, data in (**e**–**h**) by two-way ANOVA. Reported *P*-values relate to comparisons *vs*. control. Acidosis was induced by lowering the HCO_3_^–^ concentration at a fixed 5% CO_2_.

We then went on to evaluate the underlying cause of the tumor growth inhibition achieved with the 5H2.1 antibody. Colony formation of CAL51 cells was markedly reduced by incubation with the 5H2.1 antibody when applied at pH_o_ 6.8 (Fig. 6c) but unaffected at pH_o_ 7.4 (Fig. 6a). In congruence, the number of viable cells—measured with MTT assays—72 h after seeding an equal number of CAL51 cells in each well was reduced by the 5H2.1 antibody at pH_o_ 6.8 (Fig. 6d) but not at pH_o_ 7.4 (Fig. 6b).

We next explored whether the inhibitory influence of the 5H2.1 antibody on CAL51 expansion was due to effects on cell cycle progression. Our cell cycle analyses revealed that treatment with the 5H2.1 antibody caused CAL51 cells to accumulate substantively in the G2/M-phase when treated at pH_o_ 6.8 (Fig. 6f). At pH_o_ 7.4, we observed a small but significant effect of the 5H2.1 antibody that slightly reduced the number of cells in G2/M-phase and showed tendency towards S-phase accumulation (Fig. 6e).

To assess whether changes in cell survival also contribute to the reduced cancer growth during treatment with the 5H2.1 antibody, we next tested apoptosis and necrosis of CAL51 cells (Fig. 6g, h). At pH_o_ 6.8, the 5H2.1 antibody markedly increased apoptosis and showed a similar tendency towards increased necrosis (Fig. 6h), although the latter effect was not statistically significant due to low overall frequencies. At pH_o_ 7.4, the 5H2.1 antibody showed a completely opposite effect, as it lowered considerably the occurrence of both apoptosis and necrosis (Fig. 6g).

Together these findings support that pharmacological targeting of NBCn1 with inhibitory antibodies decelerates cancer growth, specifically under acidic conditions, through G2/M-phase cell cycle accumulation and enhanced apoptosis.

## Discussion

This study reports three major new discoveries. First, we provide proof-of-principle that high-affinity antibodies targeting the third extracellular loop of NBCn1 can inhibit NBCn1 activity (Figs. 1–3) and hence represent a long-sought pharmacological approach for acute and selective transporter inhibition. Second, we deliver the first direct evidence that NBCn1 is the main mechanism of net acid extrusion via Na^+^,HCO_3_^–^-cotransport from human primary breast cancer tissue (Fig. 4c), particularly in patients with regional lymph node metastasis (Fig. 4b, d, e). Third, we demonstrate that pharmacological targeting of NBCn1 can decelerate tumor growth (Fig. 5d) and cancer cell expansion (Fig. 6c, d) and cause pH-dependent G2/M-phase cell cycle accumulation (Fig. 6f) and increased apoptosis (Fig. 6h).

Interfering with net acid extrusion from cancer cells leads to intracellular acidification (Fig. 4e) that can lower cell proliferation by inhibiting RNA, DNA, and protein synthesis as well as by inhibiting cell cycle progression [52, 53]. Indeed, we previously showed that knockout of NBCn1 lowers cell proliferation in perfusion-restricted regions of murine breast cancer tissue [9, 10] and that elevated steady-state pH_i_ set by the action of Na^+^,HCO_3_^–^-cotransporters in human breast cancer tissue is associated with increased proliferation [17]. In congruence, we show here that pharmacological inhibition of NBCn1 lowers net acid extrusion from human triple-negative breast cancer tissue (Fig. 5a) and at pH_o_ 6.8 causes substantial accumulation of triple-negative human breast cancer cells in G2/M-phase of cell cycle (Fig. 6f). Consistent with these findings, the NBCn1 protein expression level in breast cancer cells was previously found to vary dynamically during cell cycle with the highest levels observed in G2/M-phase [54]. The 5H2.1 antibody showed an astoundingly different effect when applied at pH_o_ 7.4, with slight depletion of cells in G2/M-phase and tendency toward accumulation of cells in S-phase (Fig. 6e). This less conspicuous effect is in agreement with the S-phase prolongation previously reported in response to NBCn1 knockdown at pH_o_ 7.4 [54].

In a large cohort of women with breast cancer, we recently revealed that Na^+^,HCO_3_^–^-cotransport activity and NBCn1 protein expression are higher in primary breast carcinomas from patients with than without axillary lymph node metastasis [17]. In the current study, we confirm that the increased Na^+^,HCO_3_^–^-cotransport associated with lymph node-positive breast cancer is mediated exclusively via NBCn1 (Fig. 4d). We furthermore show that NBCn1 activity is elevated only in patients with macrometastases, whereas patients with micrometastases or isolated tumor cells resemble those without lymph node involvement (Fig. 4d). This observation is consistent with previous findings that NBCn1 localizes to filopodia and lamellipodia [55] where it generates pH_i_ gradients that facilitate directional cell migration [56]. The importance of NBCn1 particularly in patients with macrometastatic disease is of clear clinical importance because these patients have a worse prognosis compared with node-negative patients, and further developments in systemic therapy could improve their survival. Whereas disease-free survival is markedly reduced for patients with macrometastases in the axillary sentinel lymph nodes, the prognosis for patients with isolated tumor cells or micrometastases is close to that of patients without lymph node involvement in patients not undergoing pre-operative systemic therapy [57].

Based on patient-derived xenograft models, we show that targeting of NBCn1 can substantially inhibit tumor growth in some, but not all, triple-negative breast cancers (Fig. 5c, d). Differences in the importance of NBCn1 relative to other transporters capable of net acid extrusion (e.g., NHE1) likely explain this variation in sensitivity to NBCn1-directed therapy. Activity in cellular signaling pathways involved in post-translational activation or inhibition of NBCn1 and different metabolic signatures of tumors—including the relative dependency on fermentative glycolysis *vs*. oxidative phosphorylation—provide other likely explanations for variation in NBCn1-dependent therapeutic sensitivity. For instance, we previously found that genetic disruption of NBCn1 retards growth more substantially in murine breast carcinomas with elevated glycolytic activity and marked lactate accumulation [9, 10, 12]. It is also possible that variation between the patient-derived xenograft models in glycosylation or other post-translational modifications or in protein-protein interaction can influence the binding and functional effect of the NBCn1-targeted antibodies [58].

We find no significant effect of inhibiting NBCn1 on lactate accumulation in the culture medium harvested from human breast cancer cells (Supplementary Fig S2). Glycolytic activity and local extracellular buildup of lactate in tumors depend, in addition to cancer cell characteristics, on tissue perfusion and diffusion hindrances between the cancer cells and the blood. Using microdialysis-based sampling of interstitial solution from murine breast cancer tissue, we previously showed that NBCn1 knockout lowers lactate levels in highly glycolytic carcinogen-induced breast cancer [9] but has no effect in more moderately glycolytic ErbB2-induced breast cancer [10]. However, the lower interstitial lactate concentrations in carcinogen-induced breast cancer tissue from NBCn1 knockout mice relates to their smaller tumor sizes; [9] and consistent with our current findings (Supplementary Fig S2), the influence of NBCn1 knockout on lactate accumulation disappeared when plotted as function of tumor size [9].

Based on metastatic triple-negative breast cancer cells, we show that antibodies against NBCn1 inhibit colony formation and cell expansion (Fig. 6c, d), cause G2/M-phase cell cycle accumulation (Fig. 6f), and increase apoptosis (Fig. 6h) at low pH_o_, whereas they show no or opposite effects at physiological pH_o_ (Fig. 6a, b, e, g). Under acidic extracellular conditions, other mechanisms of net acid extrusion—particularly, transport via the Na^+^/H^+^-exchanger NHE1 [59]—are markedly inhibited, and cellular acid loading through potential H^+^ leaks will be accelerated, which can explain the stronger anti-cancer consequences of inhibiting NBCn1 at pH_o_ 6.8 compared to 7.4. The relative dependency on Na^+^,HCO_3_^–^-cotransport also typically increases when cancer cells are exposed to hypoxia [60]. Although NBCn1 is broadly expressed throughout the body [28], greater NBCn1-dependency at low pH_o_ and during hypoxia and the marked (2–7-fold) upregulation of NBCn1 during breast carcinogenesis [3, 27] support that anti-tumor therapeutic effects of targeting NBCn1 can be achieved without detrimental adverse reactions from normal tissue.

The opposite effects of inhibitory anti-NBCn1 antibodies on apoptosis at low (Fig. 6h) and normal (Fig. 6g) pH_o_ probably reflect that control of pH_i_ by Na^+^-dependent net acid extrusion comes at the cost of increased cellular Na^+^-load. Thus, although NBCn1 activity protects against excessive intracellular acidosis, it can cause cell swelling and secondary Ca^2+^-overload that may contribute to cell death [53, 61]. Most likely, lower cellular Na^+^ loading explains the protective effect of inhibitory anti-NBCn1 antibodies against cell death at neutral pH_o_, whereas excessive intracellular acidification can explain the pro-apoptotic effect at low pH_o_. The very different responses at pH_o_ 7.4 and 6.8 strengthen treatment specificity toward the tumor microenvironment, but more studies are needed to determine how they influence tumors of different metabolic activities and sizes and the targeting of primary compared to secondary tumors.

In conclusion, we show that NBCn1 can be targeted pharmacologically with functional antibodies to block net acid extrusion from breast cancer cells. The inhibitory anti-NBCn1 antibodies can lower tumor growth and—under acidic conditions—cause G2/M-phase cell cycle accumulation and elevated apoptosis in triple-negative breast cancer cells. When applied to human breast cancer tissue, we show that inhibitory anti-NBCn1 antibodies lower net acid extrusion specifically in primary cancer tissue from patients with macrometastases in the axillary lymph nodes. Together, these data strengthen the case for NBCn1 as target for human breast cancer therapy.

### Supplementary information

Supplementary Material

Reporting Checklist

## Data Availability

Data from this study are available from the corresponding author upon reasonable request. Some data may not be made available because of privacy or ethical restrictions.
