# Supplementary material for: Antibodies toward Na+,HCO3–-cotransporter NBCn1/SLC4A7 block net acid extrusion and cause pH-dependent growth inhibition and apoptosis in breast cancer
Source: Br J Cancer. 2024 Feb 3;130(7):1206–20. doi: 10.1038/s41416-024-02591-0 (PMC10991555; doi:10.1038/s41416-024-02591-0)
Supplement: Supplementary file 1 — Supplementary Material [file 41416_2024_2591_MOESM1_ESM.pdf]

Supplementary Material for

Antibodies toward Na^{+},HCO_3^{-}-cotransporter NBCn1/SLC4A7 block net acid extrusion and cause pH-dependent growth inhibition and apoptosis in breast cancer

Trine V. Axelsen[1], Claus Olesen[1], Danish Khan[1], Ali Mohammadi[1], Elena V. Bouzinova[1], Christine J. F. Nielsen[1], Marco Mele[2], Katrine R. Hauerslev[3], Helene L. Pedersen[4], Eva Balling[2], Pernille Vahl[5], Trine Tramm[5,6], Peer M. Christiansen[2,3,6], Ebbe Boedtkjer[1]

[1]Department of Biomedicine, Aarhus University, Aarhus, Denmark
[2]Department of Surgery, Randers Regional Hospital, Randers, Denmark
[3]Department of Plastic and Breast Surgery, Aarhus University Hospital, Aarhus, Denmark
[4]Department of Pathology, Randers Regional Hospital, Randers, Denmark
[5]Department of Pathology, Aarhus University Hospital, Aarhus, Denmark
[6]Department of Clinical Medicine, Aarhus University, Denmark

Supplementary Tables

Number of patients	25
Patient age (years; median, interquartile range)	70 (58-76)
Tumor size (mm; median, interquartile range)	21 (17-27)
Histological type	
Invasive ductal carcinomas	21 (84%)
Invasive lobular carcinomas	3 (12%)
Mucinous adenocarcinomas	1 (4%)
HER2 receptor status	
Normal	22 (88%)
Overexpression or gene amplification	3 (12%)
Estrogen receptor status	
Positive	22 (88%)
Negative	3 (12%)
Malignancy grade	
1	9 (36%)
2	9 (36%)
3	7 (28%)
Axillary lymph node status	
Negative	12 (48%)
Isolated tumor cells	2 (8%)
Micrometastases	3 (12%)
Macrometastases	8 (32%)
Ki67 index	
0-30% $Ki67^+$ cells	17 (68%)
35-90% $Ki67^+$ cells	8 (32%)

Supplementary Table S1. Clinical and Pathological characteristics of the breast cancer patients included in the study.

Supplementary Figures

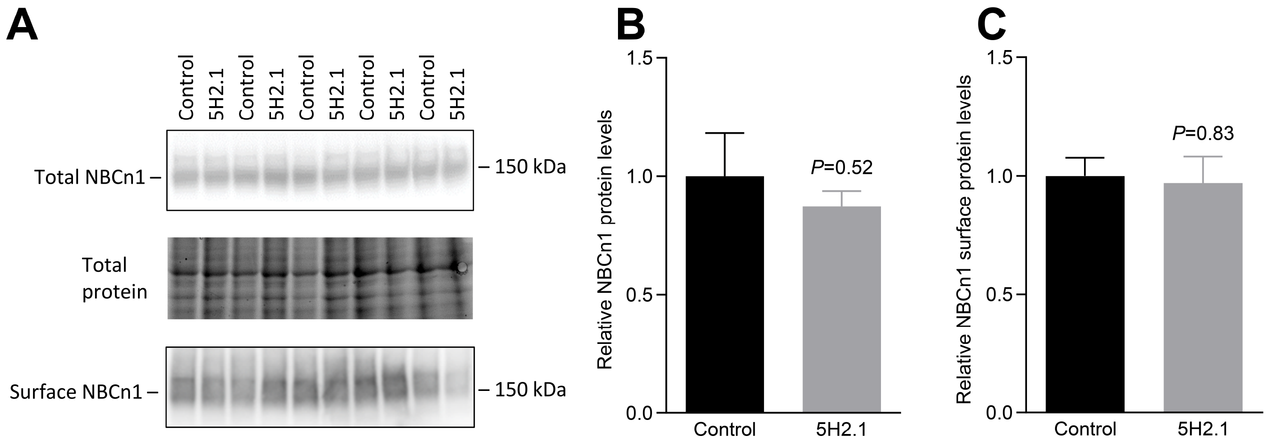

Supplementary Figure S1. NBCn1-directed inhibitory antibodies do not affect NBCn1 protein expression or membrane abundance in MCF7 breast cancer cells. **A.** Immunoblots of total protein and biotinylated surface proteins and stain-free visualization of protein loading. **B.** Total NBCn1 protein levels in MCF7 human breast cancer cells treated with 5H2.1 or vehicle for 1 hour (n=5). **C.** NBCn1 protein expression at the cell surface of MCF7 human breast cancer cells treated with 5H2.1 or vehicle for 1 hour (n=5). Surface proteins were biotinylated and then precipitated with streptavidin beads before detection with anti-NBCn1 antibody. Data were compared with unpaired, two-tailed Student's *t*-tests.

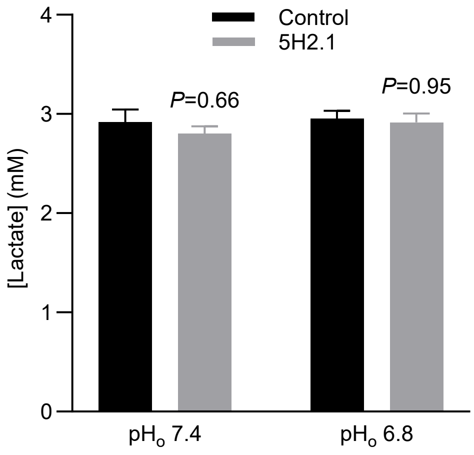

Supplementary Figure S2. Lactate production by CAL51 human breast cancer cells is unaffected by extracellular acidosis and NBCn1-directed inhibitory antibodies. The lactate concentrations in the culture medium after 24 hours of culture are shown for CAL51 cells treated with 20 nM 5H2.1 antibody or equivalent volume of vehicle (n=6). Data were compared with two-way ANOVA followed by Šidák's post-tests.
