# Supplementary material for: Antibodies toward Na+,HCO3–-cotransporter NBCn1/SLC4A7 block net acid extrusion and cause pH-dependent growth inhibition and apoptosis in breast cancer
Source: Br J Cancer. 2024 Feb 3;130(7):1206–20. doi: 10.1038/s41416-024-02591-0 (PMC10991555; doi:10.1038/s41416-024-02591-0)
Supplement: Supplementary file 2 — Reporting Checklist [file 41416_2024_2591_MOESM2_ESM.pdf]

Reporting checklist for study using laboratory animals.

Based on the ARRIVE guidelines.

Instructions to authors

Complete this checklist by entering the page numbers from your manuscript where readers will find each of the items listed below.

Your article may not currently address all the items on the checklist. Please modify your text to include the missing information. If you are certain that an item does not apply, please write "n/a" and provide a short explanation.

Upload your completed checklist as an extra file when you submit to a journal.

In your methods section, say that you used the ARRIVEreporting guidelines, and cite them as:

Percie du Sert N, Hurst V, Ahluwalia A, Alam S, Avey MT, Baker M, Browne WJ, Clark A, Cuthill IC, Dirnagl U, Emerson M, Garner P, Holgate ST, Howells DW, Karp NA, Lazic SE, Lidster K, MacCallum CJ, Macleod M, Pearl EJ, Petersen O, Rawle F, Peynolds P, Rooney K, Sena ES, Silberberg SD, Steckler T and Wurbel H. The ARRIVE Guidelines 2.0: updated guidelines for reporting animal research.

		Reporting Item	Page Number
**Essential 10**			
Study design	#1a	Give details of the groups being compared, including control groups. If no control group has been used, the rationale should be stated.	Page 13
Study design	#1b	Give details of the experimental unit (e.g., a single animal, litter, or cage of animals).	Page 13, 15
Sample size	#2a	Specify the exact number of experimental units allocated to each group, and the total number in each experiment. Also indicate the total number of animals used.	Legend of Figure 5

Sample size	#2b	Explain how the sample size was decided. Provide details of any a priori sample size calculation, if done.	Page 13
Inclusion and exclusion criteria	#3a	Describe any criteria used for including or excluding animals (or experimental units) during the experiment, and data points during the analysis. Specify if these criteria were established a priori. If no criteria were set, state this explicitly.	Page 13
Inclusion and exclusion criteria	#3b	For each experimental group, report any animals, experimental units, or data points not included in the analysis and explain why. If there were no exclusions, state so.	Page 13
Inclusion and exclusion criteria	#3c	For each analysis, report the exact value of n in each experimental group.	Legend of Figure 5
Randomisation	#4a	State whether randomisation was used to allocate experimental units to control and treatment groups. If done, provide the method used to generate the randomisation sequence.	Page 13
Randomisation	#4b	Describe the strategy used to minimise potential confounders such as the order of treatments and measurements, or animal/cage location. If confounders were not controlled, state this explicitly.	Page 13
Blinding	#5	Describe who was aware of the group allocation at the different stages of the experiment (during the allocation, the conduct of the experiment, the outcome assessment, and the data analysis).	Page 13
Outcome measures	#6a	Clearly define all outcome measures assessed (e.g., cell death, molecular markers, or behavioural changes).	Page 13
Outcome measures	#6b	For hypothesis-testing studies, specify the primary outcome measure, i.e., the outcome	Page 13

		measure that was used to determine the sample size.	
Statistical methods	#7a	Provide details of the statistical methods used for each analysis, including software used.	Page 13, 15 and legends
Statistical methods	#7b	Describe any methods used to assess whether the data met the assumptions of the statistical approach, and what was done if the assumptions were not met.	Page 15
Experimental animals	#8a	Provide species-appropriate details of the animals used, including species, strain and substrain, sex, age or developmental stage, and, if relevant, weight.	Page 13
Experimental animals	#8b	Provide further relevant information on the provenance of animals, health/immune status, genetic modification status, genotype, and any previous procedures.	Page 13
Experimental procedures	#9a	For each experimental group, including controls, describe the procedures in enough detail to allow others to replicate what was done, how it was done, and what was used.	Page 13
Experimental procedures	#9b	Timing and frequency of procedures	Page 13
Experimental procedures	#9c	Where procedures were carried out (including detail of any acclimatisation periods).	Page 12-13
Experimental procedures	#9d	Rationale for procedures	Page 12
Results	#10a	For each experiment conducted, including independent replications, report summary/descriptive statistics for each experimental group, with a measure of variability where applicable (e.g., mean and SD, or median and range).	
Figure 5 and legend			
Results	#10b	If applicable, for each experiment conducted, including independent replications, report the effect size with a confidence interval.	Figure 5

Recommended set

Abstract	#11	Provide an accurate summary of the research objectives, animal species, strain and sex, key methods, principal findings, and study conclusions.	Page 2
Background	#12a	Include sufficient scientific background to understand the rationale and context for the study, and explain the experimental approach.	Page 3-4
Background	#12b	Explain how the animal species and model used address the scientific objectives and, where appropriate, the relevance to human biology.	Page 3-4
Objectives	#13	Clearly describe the research question, research objectives and, where appropriate, specific hypotheses being tested.	Page 3-4
Ethical statement	#14	Provide the name of the ethical review committee or equivalent that has approved the use of animals in this study and any relevant licence or protocol numbers (if applicable). If ethical approval was not sought or granted, provide a justification.	Performed by Charles River
Housing and husbandry	#15	Provide details of housing and husbandry conditions, including any environmental enrichment.	Performed by Charles River
Animal care and monitoring	#16a	Describe any interventions or steps taken in the experimental protocols to reduce pain, suffering, and distress.	Performed by Charles River
Animal care and monitoring	#16b	Report any expected or unexpected adverse events.	Page 13
Animal care and monitoring	#16c	Describe the humane endpoints established for the study, the signs that were monitored, and the frequency of monitoring. If the study did not set humane endpoints, state this.	Page 13
Interpretation/scientific implications	#17a	Interpret the results, taking into account the study objectives and hypotheses, current	Page 18-19

		theory, and other relevant studies in the literature.	
Interpretation/scientific implications	#17b	Comment on the study limitations, including potential sources of bias, limitations of the animal model, and imprecision associated with the results.	Page 21-24
Generalisability/translation	#18	Comment on whether, and how, the findings of this study are likely to generalise to other species or experimental conditions, including any relevance to human biology (where appropriate).	Page 21-24
Protocol registration	#19	Provide a statement indicating whether a protocol (including the research question, key design features, and analysis plan) was prepared before the study, and if and where this protocol was registered.	Page 12
Data access	#20	Provide a statement describing if and where study data are available.	Page 26
Declaration of interests	#21a	Declare any potential conflicts of interest, including financial and nonfinancial. If none exist, this should be stated.	Page 26
Declaration of interests	#21b	List all funding sources (including grant identifier) and the role of the funder(s) in the design, analysis, and reporting of the study.	Page 26

None The ARRIVE checklist is distributed under the terms of the Creative Commons Attribution License CC-BY. This checklist can be completed online using https://www.goodreports.org/, a tool made by the EQUATOR Network in collaboration with Penelope.ai
